# Magnesium Deficiency Induced Global Transcriptome Change in *Citrus sinensis* Leaves Revealed by RNA-Seq

**DOI:** 10.3390/ijms20133129

**Published:** 2019-06-26

**Authors:** Lin-Tong Yang, Yang-Fei Zhou, Yan-Yu Wang, Yan-Mei Wu, Xin Ye, Jiu-Xin Guo, Li-Song Chen

**Affiliations:** 1 Institute of Plant Nutritional Physiology and Molecular Biology, Fujian Agriculture and Forestry University, Fuzhou 350002, China; 2 College of Resources and Environment, Fujian Agriculture and Forestry University, Fuzhou 350002, China; 3 Fujian Provincial Key Laboratory of Soil Environmental Health and Regulation, Fujian Agriculture and Forestry University, Fuzhou 350002, China

**Keywords:** Mg deficiency, *Citrus sinensis*, transcriptome, cellular transport, signal transduction

## Abstract

Magnesium (Mg) deficiency is one of the major constraining factors that limit the yield and quality of agricultural products. Uniform seedlings of the *Citrus sinensis* were irrigated with Mg deficient (0 mM MgSO_4_) and Mg sufficient (1 mM MgSO_4_) nutrient solutions for 16 weeks. CO_2_ assimilation, starch, soluble carbohydrates, TBARS content and H_2_O_2_ production were measured. Transcriptomic analysis of *C. sinensis* leaves was performed by Illumina sequencing. Our results showed that Mg deficiency decreased CO_2_ assimilation, but increased starch, sucrose, TBARS content and H_2_O_2_ production in *C. sinensis* leaves. A total of 4864 genes showed differential expression in response to Mg deficiency revealed by RNA-Seq and the transcriptomic data were further validated by real-time quantitative PCR (RT-qPCR). Gene ontology (GO) enrichment analysis indicated that the mechanisms underlying Mg deficiency tolerance in *C. sinensis* may be attributed to the following aspects: (a) enhanced microtubule-based movement and cell cycle regulation; (b) elevated signal transduction in response to biotic and abiotic stimuli; (c) alteration of biological processes by tightly controlling phosphorylation especially protein phosphorylation; (d) down-regulation of light harvesting and photosynthesis due to the accumulation of carbohydrates; (e) up-regulation of cell wall remodeling and antioxidant system. Our results provide a comprehensive insight into the transcriptomic profile of key components involved in the Mg deficiency tolerance in *C. sinensis* and enrich our understanding of the molecular mechanisms by which plants adapted to a Mg deficient condition.

## 1. Introduction

Magnesium (Mg) is an essential macronutrient for plant growth and production. As the eighth most abundant element in the Earth’s crust, soil Mg originates from weathering of minerals containing Mg, such as serpentine, bishopvillite, calcite, dolomite, magnesite, periclase, talcum and diopside. Due to high intensity agriculture, unawareness of the need for Mg fertilizer application and rainy climate, Mg deficiency is a frequently observed nutrient disorder in agricultural production, including the *Citrus* industry [1,2]. Understanding the physiological and molecular mechanisms underlying plant adaptation to Mg deficiency is of theoretical and practical value for high-quality and high-yield agricultural production.

According to published literature, the best recognized physiological functions of Mg in plants are the formation of chlorophyll pigments [3], the stabilization of specific structural motifs for both nucleic acids and their complexes with cognate ligands [4], the proper grana stacking of thylakoid membranes in chloroplast [5], the activation of carboxylation activity of Rubisco in leaves [6], the regulation of the activity of enzymes in the Calvin cycle [7] and the allosteric activation of protein complexes [8]. Chlorophyll a fluorescence analysis indicated that Mg deficiency also significantly decreased the oxygen evolution, electron transport and efficiency of photochemical energy conversion by photosystem II (PSII) in *Citrus* and maize leaves [9,10]. Due to the key role of Mg in phloem loading of sucrose and photosynthate partitioning between source and sink organs under Mg deficiency [11,12], the dysfunction of photosynthate transport consequently led to the accumulation of carbon in resource leaves before the appearance of chlorosis and necrosis, and resupply of Mg rapidly enhanced the sucrose export through phloem from resource leaves [13]. Such quick recovery of the sucrose export regardless of light condition indicated that enhancement of sucrose export after Mg resupply is only related to Mg availability [3]. Research found that the decreased Mg-ATP concentration at the phloem-loading sites is the major reason for inhibiting sucrose transport from the Mg-deficient source leaves [14]. This suggested that trans-membrane transportation carrier or channel-dependent phloem-loading of sucrose was prompted by Mg-ATP complex and Mg deficiency led to decreased H^+^-ATPase activity and a reduced proton gradient which energizes active phloem loading.

Magnesium is essential for the synthesis of proteins, as it is needed for the assembly of the ribosomal subunits and the uptake of nitrogen in soil [15,16]. The quality of faba beans, with regard to the protein concentration, was reduced in response to the magnesium deficiency treatment [17]. Zhao et al. [10] reported that Mg deficiency significantly inhibited the plant growth and decreased the activities of nitrate reductase (NR), sucrose-phosphate synthase (SPS) and phosphoenolpyruvate carboxylase (PEPC), and the synthesis of chlorophyll and protein in maize. Our previous study also found that Mg deficiency decreased the content of free amino acids in *C. sinensis* leaves [18] and the total soluble protein in *C. sinensis*, *C. grandis* and *C. reticulata* leaves [1,9,19]. Both the proteomic and transcriptomic analysis suggested that the decrease of total protein levels was probably due to the upregulated degradation of dysfunctional protein caused by excess reactive oxygen species (ROS) rather than the decreased biosynthesis of protein [1,19]. Besides altered protein metabolism, Mg deficiency also has impacts on several biological processes, such as antioxidant metabolism, cell wall reconstruction and secondary metabolism.

ROS, including singlet oxygen (^1^O_2_), superoxide anion (O_2_^−^), hydrogen peroxide (H_2_O_2_) and hydroxyl radical (HO^−^), are partially reduced or excited forms of atmospheric oxygen and can be produced in nearly every sub-cellular compartment. They function in cells as signaling molecules as well as the unavoidable toxic byproducts of aerobic metabolism [20]. A feedback inhibition mechanism via sugar accumulation leads to lower utilization of the photosynthetic electron transport chain, as indicated by decreased CO_2_ assimilation. Imbalance between light capture and utilization typically triggers the production of ROS in Mg-deficient plants [21]. In fact, ROS burst and up-regulation of the antioxidant system in Mg-deficient leaves was observed in mulberry [22], *Mentha* [23], maize [24], pepper [25], rice [26] and *Citrus* [18].

The plant defense response is associated with the activation of the general phenylpropanoid pathway and induction of lignin biosynthesis [27]. Our previous studies revealed that the content of metabolites, the expression levels of gene or enzymes related to lignin biosynthesis, such as total phenolics, cytochrome P450, phenylalanine ammonia-lyase (PAL), guaiacol peroxidase (GPX), were upregulated by Mg deficiency in *Citrus* [18,19]. Except for ROS, information about biological regulation and signal transduction responding to Mg deficiency in plants is scarce, although some reports indicated that Mg deficiency resulted in the expression changes of some genes, including ethylene biosynthesis, protein degradation, abscisic acid (ABA) response and circadian rhythm [1,3,28]. Hermans et al. reported that Mg deficiency triggered ABA signaling, with half of the upregulated genes in leaves being ABA-responsive, even though no change in ABA content was observed [28]. Another study found that Mg deficiency increased the content of ethylene and the expression levels of some genes encoding enzymes (1-aminocyclopropane-1-carboxylic acid synthase 2; 7; 8; 11) in the ethylene biosynthetic pathway in model plant *Arabidopsis* [29], implying that ethylene may play a key role in response to Mg status in plants [30].

*Citrus* is an evergreen fruit tree that is cultivated in tropical and sub-tropical areas, most of which have a heavy rainfall and high temperature climate. According to our previous survey of 319 *Citrus* orchards in Fujian province, China, soil acidification was a major problem in these orchards, with an average pH of 4.34. In addition, 77.4% of soil samples were sub-optimum in exchangeable Mg and 35.6% of leaves were Mg deficient [2]. We have thus far investigated the metabolism of organic acid [31], photosynthesis and the antioxidant system [9], proteomic profile [1], cDNA-AFLP analysis [19] and the metabolism of key metabolites [18] in *Citrus* plants under Mg deficiency. In order to gain new insight into the molecular mechanism of plant adaption to low Mg condition and ensure green and sustainable development of agriculture, we used the RNA-Seq technique to investigate transcriptomic profile of *C. sinensis* under long-term Mg deficiency in this study.

## 2. Results

### 2.1. Effects of Mg Deficiency on Dry-Weight (DW), Mg Content, Leaf Gas Exchange Parameters and Soluble Sugar Content of C. sinensis

**Figure 1:**
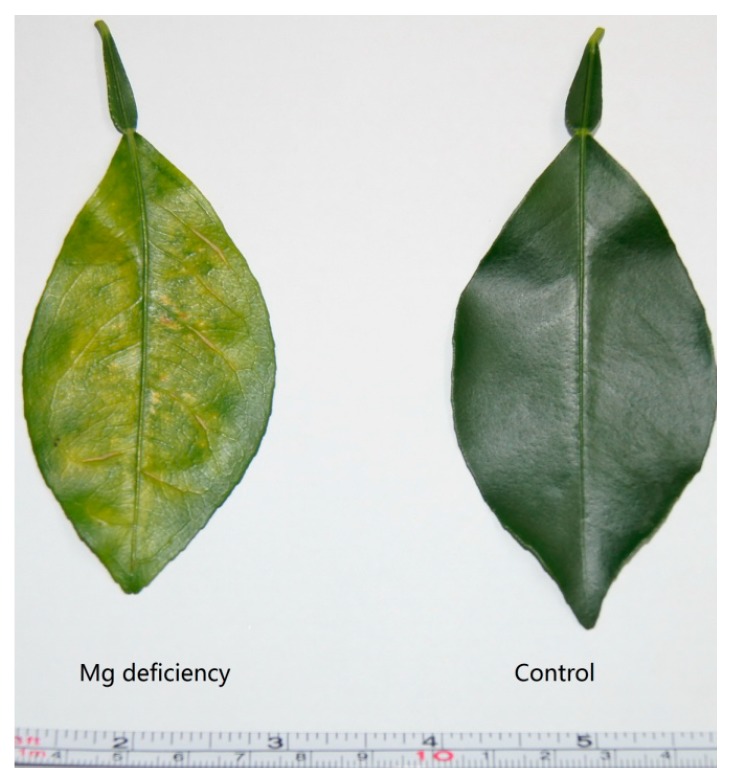
Symptom of Mg deficiency in *C. sinensis* leaf.

**Figure 2:**
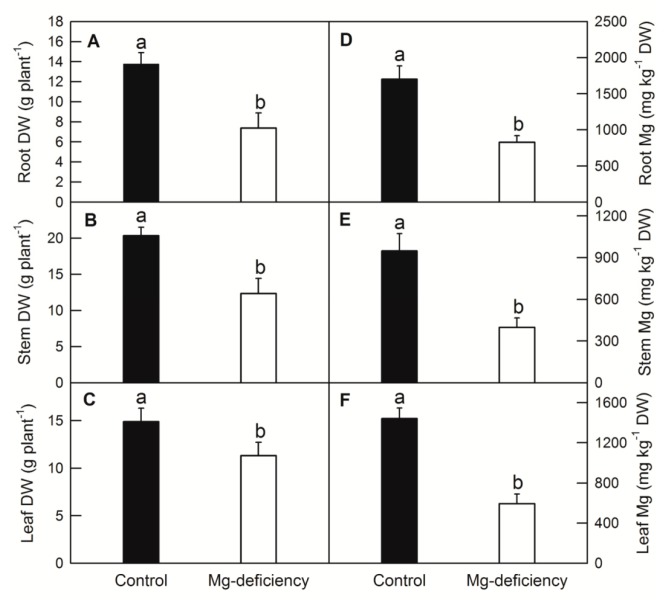
Effects of Mg deficiency on plant dry weight (DW; root, (**A**); stem, (**B**); shoot, (**C**)) and Mg content (root, (**D**); stem, (**E**); leaf, (**F**)) in *C. sinensis* seedlings. Bars represent means ±SD (*n* = 5 for Mg content or 10 for plant DW). Difference among the treatments was analyzed by student’s *t*-test. Different letters indicate a significant difference at *p* < 0.05.

**Figure 3:**
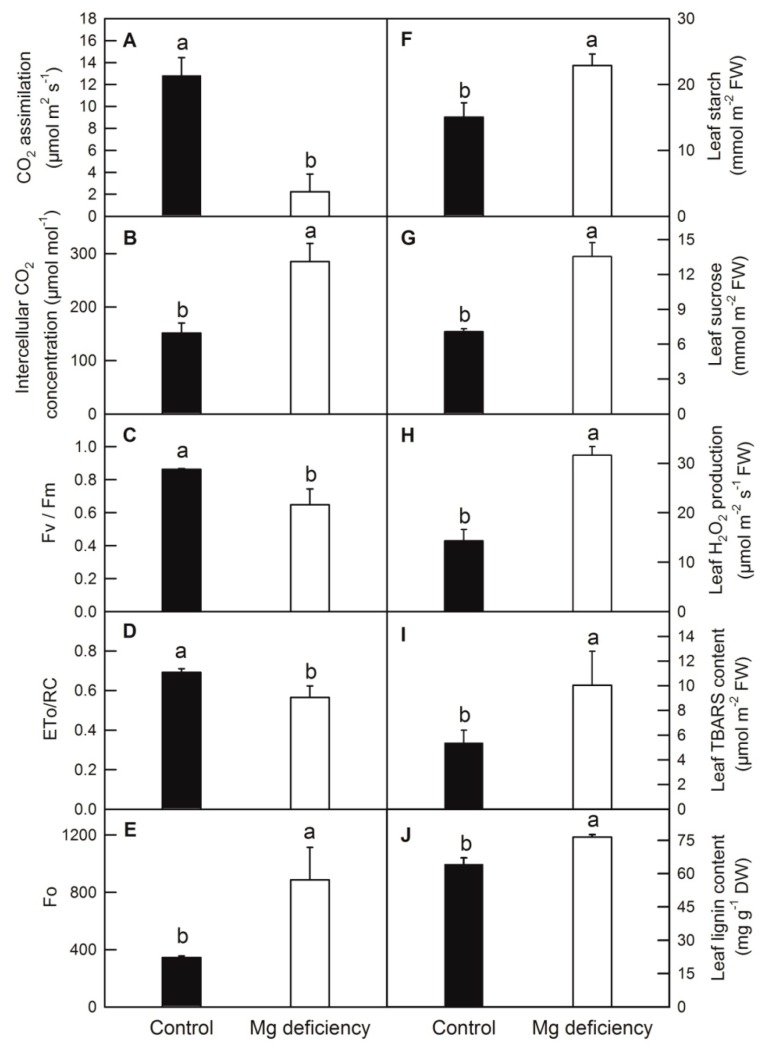
Effects of Mg deficiency on CO_2_ assimilation (**A**), intercellular CO_2_ (**B**), maximum quantum yield of primary photochemistry (Fv/Fm) (**C**), electron transport flux per reaction center at t = 0 (ET_o_/RC) (**D**), minimum fluorescence (F_o_) (**E**), leaf starch (**F**), sucrose (**G**), H_2_O_2_ production (**H**), TBARS (**I**) and lignin (**J**) content in *C. sinensis*. Bars represent means ±SD (*n* = 4). Difference among the treatments was analyzed by student’s *t*-test. Different letters indicate a significant difference at *p* < 0.05.

Mg deficiency (-Mg) leads to a symptom of chlorosis and protruding veins in *C. sinensis* leaf (Figure 1). Mg deficiency dramatically decreased the DW and Mg content of root (Figure 2A,D), stem (Figure 2B,E) and leaf (Figure 2C,F) in *C. sinensis*. Mg deficiency decreased CO_2_ assimilation and increased the intercellular CO_2_ concentration in *C. sinensis* leaves (Figure 3A,B). Chlorophyll a fluorescence measurement indicated that Mg deficiency decreased the maximum quantum yield of primary photochemistry (F_v_/F_m_), electron transport flux per reaction center at t = 0 (ET_o_/RC) and increased the minimum fluorescence (F_o_) (Figure 3C–E). Due to the fundamental role of Mg in photosynthate transport, Mg deficiency impaired sucrose transport from source leaves to sink tissues, and thus both starch and sucrose were higher in -Mg leaves than in the control ones (Figure 3F,G).

### 2.2. Mg Deficiency Increased Reactive Oxygen Species Generation, Membrane Lipid Peroxidation and Lignin Content

Mg deficiency significantly increased reactive oxygen species stress and membrane lipid peroxidation, revealed by the increased production of H_2_O_2_ and TBARS content, respectively (Figure 3H,I). Moreover, in order to testify whether accumulated photosynthates could induce lignin biosynthesis, the lignin content was measured. Data showed that Mg deficiency could actually increase the lignin content in *C. sinenesis* leaves (Figure 3J).

### 2.3. RNA-Seq, De Novo Assembly, Transcripts Annotation and Differentially Expressed Genes (DEGs) Identification

**Figure 4:**
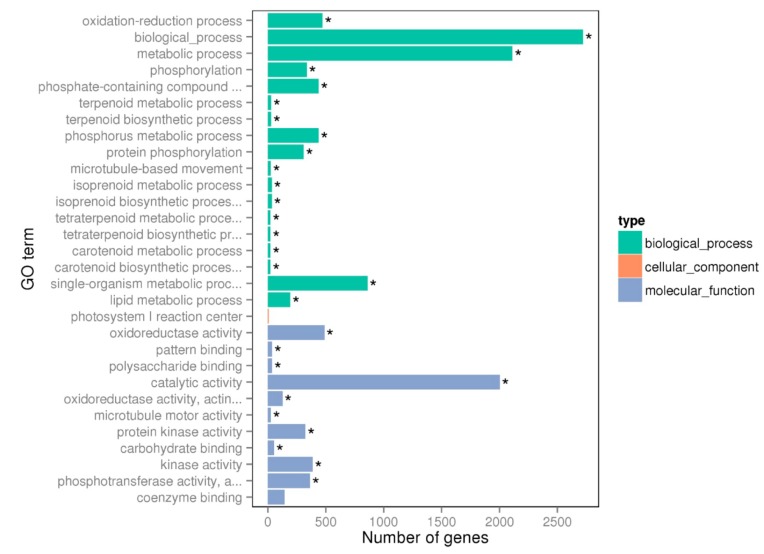
Gene ontology (GO) term enrichment analysis of differentially expressed genes (DEGs) in *C. sinensis* leaves. * Asterisk means DEGs were significantly enriched in this GO term.

To explore the transcriptomic change of *C. sinensis* leaves in response to Mg deficiency, the control and Mg deficiency libraries were constructed by using NEBNext^®^ Ultra™ RNA Library Prep Kit for Illumina^®^ (NEB, Beverly, MA, USA) and cDNA fragments of preferentially 150~200 bp in length were purified with AMPure XP magnetic beads (Beckman Coulter Genomics, Danvers, MA, USA). Each treatment contained two biological replicates and each biological replicate comprised mixed samples from five different plants. We did not sequence three biological replicates considering that we used two treatments of *C. sinensis* and the data from the control and the Mg-deficient samples could mutually corroborate each other. After cluster generation, the library preparations were sequenced on an Illumina Hiseq platform and 125 bp/150 bp paired-end reads were generated. By removing reads containing adapter, reads containing ploy-N and low quality reads from raw data, clean reads were eventually obtained. At the same time, Q20 and GC content in the clean data was calculated. The obtained libraries contained total reads ranging from 48,980,872 to 63,560,528, clean reads ranging from 46,926,606 to 60,296,780, clean bases ranging from 6.71 G to 9.04 G and Q20 values ranging from 96.27 to 97.23 (Table 1). Genome mapping revealed that total mapped reads, multiple mapped reads and uniquely mapped reads ranged from 35,701,067 to 44,649,336, 1,256,345 to 1,515,278, and 32,854,616 to 43,134,058, respectively. Here, 71.03% to 73.43% of the clean reads were uniquely mapped to the *Citrus* genome, which was consistent with our previous transcriptomic analysis (Table 1) [32]. The gene expression levels represented by FPKM showed that the proportion of 0~1 FPKM interval was nearly 50%, followed by 3~15, 15~60 and 1~3 FPKM intervals (Table 2). Interestingly, Mg deficiency increased the proportion of relatively high abundance genes (15~16, >60 FPKM interval) and decreased the proportion of relatively low abundance genes (0~1 FPKM interval). According to the criteria of log_2_(fold change) > 1 and FDR < 0.05, a total of 2176 downregulated and 2688 upregulated genes were identified as DEGs that responded to Mg deficiency in *C. sinensis* leaves (Tables S1 and S2). Gene ontology (GO) enrichment analysis showed that 28 GO terms with a corrected *p* < 0.05 were significantly enriched by using the method of Wallenius non-central hyper-geometric distribution (Table S3) [33]. In order to clarify which biological pathway was involved in the Mg deficiency tolerance in *C. sinensis*, 18 GO terms out of 28 enriched GO terms, which belong to the term type of biological process, would be the main topic of subsequent discussion (Figure 4).

### 2.4. Validation of RNA-Seq Results by Real-Time Quantitative PCR (RT-qPCR)

**Figure 5:**
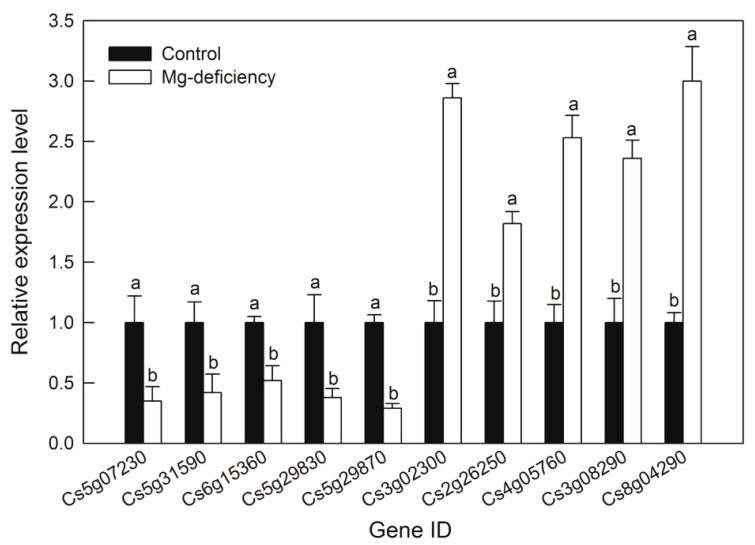
Relative expression levels of ten DEGs under Mg deficiency in *C. sinensis* leaves. Cs5g07230: cytochrome P450; Cs5g31590: tRNA delta (2)-isopentenyl pyrophosphate transferase; Cs6g15360: dehydration-responsive element-binding protein 1B; Cs5g29830: MYB4; Cs5g29870: ethylene-responsive transcription factor 1B; Cs3g02300: helicase SEN1; Cs2g26250: calcium-transporting ATPase 2; Cs4g05760: WRKY50; Cs3g08290: isoflavone reductase; Cs8g04290: transposon protein. Bars represent means ±SD (*n* = 3). Difference among the treatments was analyzed by student’s *t*-test. Different letters indicate a significant difference at *p* < 0.05.

In order to test the RNA-Seq result, primer pairs of cytochrome P450 (Cs5g07230), tRNA delta(2)-isopentenyl pyrophosphate transferase (Cs5g31590), dehydration-responsive element-binding protein 1B (Cs6g15360), MYB4 (Cs5g29830), ethylene-responsive transcription factor 1B (Cs5g29870), helicase SEN1 (Cs3g02300), calcium-transporting ATPase 2 (Cs2g26250), WRKY50 (Cs4g05760), isoflavone reductase (Cs3g08290) and transposon protein (Cs8g04290) were generated and RT-qPCR was carried out. The data of RT-qPCR and RNA-Seq were completely consistent with each other, indicating that RNA-Seq is a robust and reliable method to isolate and identify Mg-deficiency-respondent DEGs in *C. sinensis* (Figure 5).

## 3. Discussion

### 3.1. Mg Deficiency Increased Microtubule-Based Movement (GO: 0007018)

Mg plays a vital role in the transport of photosynthate through phloem from source tissue to sink tissue in plants [34]. Among the photosynthates measured in Mg-deficient plants, sucrose was the most pronounced one that accumulated in old matured leaves and resupplying Mg to Mg-deficient plants apparently restored phloem export of sucrose within 12 h [12]. Moreover, evidence suggested that Mg-ATP is the major complex of ATP in biological system and a fall in concentration of Mg-ATP at the phloem-loading site is the major reason for the inhibited sucrose loading in Mg-deficient leaves [14,35]. In the current study, Mg deficiency conspicuously influenced the gene expression level of kinesins with 22 kinesin-encoded genes upregulated and three downregulated (Table S4). According to the available literature, kinesin is a two-headed motor protein that powers organelle transport along microtubules even in the absence of attachment to a cargo. Further study showed that two heads of kinesin can force the long distance movement along microtubule by hydrolyzing ATP molecules [36]. This gave rise to the possibility that Mg might perform its role by interacting with ATP to form Mg-ATP complex, which was subsequently utilized by kinesin to force cargo movement along microtubule. However, whether the lack of Mg-ATP caused by the depletion of Mg or the accumulation of cargo such as sucrose in *C. sinensis* leaves is the main reason for the up-regulation of the kinesin genes remains further investigation. Among the differentially expressed kinesin genes, some of them were associated with cell cycle regulation, such as genes encoding chromosome-associated kinesin (KIF4) and kinesin (centromere protein) like heavy chain-like protein (Table S4). This indicated that Mg deficiency may also disturb normal cell replication and proliferation in *C. sinensis* leaves.

### 3.2. Mg Deficiency Affected Carbohydrate Binding Activities (GO: 0030246)

The accumulation of carbohydrate in the mature leaves more or less impacted upstream and downstream biological process such as starch hydrolysis, amino acid metabolism, secondary compounds synthesis, plasma membrane transport activity [9,13,18,37]. As we expected, Mg deficiency influenced the expression level of an amount of genes related to carbohydrate binding activities compared to control ones (Table S5). Among these DEGs, ten genes were downregulated and 44 genes were upregulated by Mg deficiency. Interestingly, all the gene expression levels of five cold-inducible wall-associated kinases (*WAKs*) and 11 *WAK*-like genes were higher in the Mg-deficient leaves than in the control ones. Available evidence showed that WAKs belong to a protein family that was implicated primarily in signal transduction in host response to pathogen attack and particular in the perception of pectin-containing fragments [38,39,40]. Furthermore, Sivaguru et al. [41] found that *WAK1* was involved in aluminum stress in *Arabidopsis* and thus may participate in heavy metal response. This result indicated that *WAKs*/*WAK*-like genes might participate in a global response to environmental stimuli including Mg deficiency. Similar to WAKs, Lectin-domain containing receptor protein kinase (LecLPK) is believed to play crucial roles in saccharide signaling as well as stress perception in plants [42]. Expression of the *AtLecLPK1* was strongly induced by ABA, methyl jasmonate (MJA), salicylic acid (SA) and stress treatments including salt stress in *Arabidopsis* [43]. Here, three upregulated *LecLPKs* were isolated from -Mg *C. sinensis* leaves (Table S5 (Supplementary Materials)). This result corroborated that Mg deficiency, as a nutrient disorder, like other environment stimuli, could trigger a cascade of events along signal transduction and stress perception. Small glutamine-rich tetratricopeptide repeat (TPR)-containing protein (SGT) alpha (SGTA) belongs to a family of molecular co-chaperone proteins that harbored a TPR motif, acting as a protein–protein interaction module, which was capable of facilitating interaction with a diverse range of client proteins in animal [44]. In plants, except for a reference reporting *SGT* beta subunit that was associated with chromium (Cr) tolerance in spring wheat, limited data about *SGT* led to further studies on the function of this gene [45]. The upregulated *SGTA* in -Mg *C. sinensis* leaves means that *SGTA* may also respond to nutrient disorder in plants. Furthermore, among DEGs enriched in carbohydrate binding activities, genes encoding 2-acetamido-2-deoxy-d-galactose-binding seed lectin 2, 3-hexulose-6-phosphate isomerase, chloroplast post-illumination chlorophyll fluorescence increase protein isoform 1(fragment), glucoamylase and uncharacterized ribonuclease sll1290, which are implicated in plant defense against pathogen and herbivory [46], assimilating endogenous gaseous formaldehyde [47], chlororespiration in protecting plastoquinone (PQ) from over-reduction and thereby also photosystem (PS) I from photoinhibition [48], decomposition of starch [49], and posttranscriptional regulation of gene expression [50], respectively, were downregulated by Mg deficiency in *C. sinensis* leaves. These RNA-Seq data were consistent with our physiological and chlorophyll a fluorescence measurement, which showed that -Mg leaves accumulated a higher content of starch and suffered more severe photoinhibition (Figure 2C–E). Brassinosteroid (BR) insensitive 1-associated receptor kinase, also known as leucine-rich repeat receptor like kinase, positively regulated plant growth, development and response to biotic and abiotic stresses by directly binding to the BR ligand, triggering a signal cascade in the cytoplasm that leads to the transcription of BR-responsive genes [51]. The upregulated gene expression levels of several BR insensitive 1-associated receptor kinases in *C. sinensis* leaves might imply that BRs participated in the response to Mg deficiency in *Citrus* plants (Table S5).

### 3.3. Mg Deficiency Affected Phosphorus Metabolic Process (GO:0006793, GO:0016310 and GO:0006468)

Phosphorylation, especially protein phosphorylation, is a crucial biological regulation in plant growth and development. Our transcriptomic analysis revealed that Mg deficiency altered the expression of 439 genes involved in phosphorus metabolic process, of which, 337 and 309 genes were related to phosphorylation process and protein phosphorylation, respectively (Table S6).

Protein phosphorylation is the most abundant post-translational modification (PTM) of functional proteins and a key regulatory step in the eukaryotic signal transduction [52]. It was supposed to govern various processes such as CO_2_ assimilation [53], chromatin stabilization [54], Ca signaling [55], hormone signaling [56], protein trafficking [57], protein turnover [58], photosynthate transport [59], circadian regulation [60] and pathogen-perception [61]. Here, among the 309 DEGs involved in protein phosphorylation (Table S6), 234 and 75 DEGs were upregulated and downregulated in *C. sinensis* leaves by Mg deficiency, respectively. Interestingly, more than one-third of the 309 DEGs belong to the serine-threonine protein kinase family, which is consistent with the concept that most phosphorylation events occur in serine or threonine residues in plants [52]. Meanwhile, as we expected, the conserved genes involved in protein phosphorylation, such as Protein kinase APK1B (*PK*), calcium-dependent protein kinase (*CDPK*), CBL-interacting protein kinase (*CIPK*), mitogen-activated protein kinase (*MAPK*)/kinase (*MAPKK*) and LRR protein kinase were differentially regulated by Mg deficiency. This result indicated that the sequential activation or inactivation of the downstream cascade may result in the changed processes of cell division, growth, differentiation, programmed cell death and environmental stress response [62]. Such expression alteration of the genes related to protein phosphorylation was also observed in *Citrus* and tobacco treated with boron deficiency [63,64], *Poncirus trifoliata* and soybean treated with phosphorus deficiency [65,66], rice treated with calcium deficiency [67], and cassava treated with low-temperature [68]. Evidence showed that G-type lectin S-receptor-like serine/threonine protein kinase (GsSRK) was a positive regulator of tolerance to salt stress in wild soybean, *Gossypium barbadense* and *Arabidopsis* [68,69]. Additionally, Vaid et al. [70] also found that GsSRKs are highly induced under abiotic stress conditions including heat, drought, salt, and cold in *Arabidopsis* and rice. Here, 18 differentially expressed *GsSRKs* were isolated from *C. sinensis* leaves and most of them were upregulated by Mg deficiency (Table S6). Besides protein phosphorylation, various genes involved in substrate phosphorylation were also altered in *C. sinensis* leaves by Mg deficiency (Table S6). Multidrug resistance-associated protein 2,6 (MRP2,6) can use the energy generated by the hydrolysis of Mg-ATP to facilitate the transmembrane movement of a variety of small molecules [71]. Here, we found that the expression level of *MRP2,6* was downregulated by Mg deficiency in *C. sinensis* leaves (Table S6). This may further confirm that Mg deficiency hinders the transport of cargos including sugar due to the decreased Mg-ATP as we mentioned above. Interestingly, the gene encoding phototropin-1, which is an important mediator in the phototropic growth and stomata on-off in plants, was also downregulated by Mg deficiency (Table S6) [72], indicating that *C. sinensis* can self-adapt to relative high photo-radiation to ameliorate the photo-oxidative and photo-inhibition damage when the efficiency of photosynthetic electron transport and photosynthesis was impaired by Mg deficiency [9,19]. Furthermore, the genes encoding clathrin interactor 1, cyanidin-3-O-glucoside 2-O-glucuronosyltransferase, gamma-glutamyl hydrolase, lipase, L-lactate dehydrogenase, pyruvate kinase (cytosolic), ribonucleoside-diphosphate reductase small chain, etc., were upregulated in *C. sinensis* leaves by Mg deficiency (Table S6). The alteration of these genes implies that the endocytic pathway, glycolysis, anthocyanins modification, lipid metabolism, chromatin organization, etc., may be altered in response to Mg deficiency [31,73,74]. Genes encoding the proteins mentioned above should be further validated to understand their physiological role in tolerance to Mg deficiency in the future study.

### 3.4. Mg Deficiency Affected the Genes Involved in Isoprenoid, Terpenoid and Carotenoid Metabolic Process (GO:0006720, GO:0006721 and GO:0016116)

Isoprenoids are a fascinating family of compounds derived from the C5 precursor isopentenyl diphosphate (IPP) and dimethylallyl diphosphate (DMAPP), which contain primary and secondary metabolite [75]. Isoprenoids function in respiration (ubiquinone), photosynthesis (carotenoid, chlorophyll, tocopherol, plastoquinone), membrane architecture (sterol) and growth regulation (brassinosteroid, cytokinin, gibberellin, abscisic acid, strigolactone), whereas the secondary compounds mediate important interaction between plants and their environment [76]. In plants, there are two separate and biochemically different IPP biosynthesis pathways, named the mevalonic acid (MVA) and methylerythritol 4-phosphate (MEP) pathway. The MEP pathway simultaneously produces both IPP and DMAPP from pyruvate and glyceraldehyde 3-phosphate (GAP) in the plastids, whereas the MVA pathway synthesizes IPP from acetyl-CoA in the cytoplasm [77,78]. Here, we found that genes encoding geranylgeranyl pyrophosphate synthase (3 genes), probable 1-deoxy-d-xylulose-5-phosphate synthase (chloroplastic, 2 genes), putative geranyl diphosphate synthase (1 gene) and 2-C-methyl-d-erythritol 2,4-cyclodiphosphate synthase (chloroplastic, 1 gene), which involved in MEP pathway, were downregulated in *C. sinensis* leaves by Mg deficiency (Table S7). Furthermore, the generation of certain isoprenoid derivatives such as chlorophyll, ubiquinone, lycopene and zeaxanthin, may also be hindered by Mg deficiency due to the downregulated gene expression of protoporphyrinogen oxidase (chloroplastic), electron transfer flavoprotein-ubiquinone oxidoreductase, lycopene epsilon cyclase (chloroplastic), lycopene beta cyclase (chloroplastic/chromoplastic), zeaxanthin epoxidase (chloroplastic) (Table S7). Interestingly, the expression level of glycerol-3-phosphate dehydrogenase (*SDP6*, mitochondrial) was upregulated by Mg deficiency, which could result in the increasing conversion of dihydroxyacetone phosphate (DHAP) to glycerol-3-phosphate (G3P) and decrease DHAP to GAP shunt [79]. This result may further support the point that Mg deficiency decreased MEP-mediated isoprenoids biosynthesis in *C. sinensis* leaves. Such down-regulation of the MEP pathway was also observed in poplar leaves under UV-B radiation [80]. The reduction in the MEP pathway under Mg deficiency might result from elevated intercellular CO_2_ (Figure 3A), which may enhance higher consumption rate of cytosolic PEP (phosphoenolpyruvate) through increased PEP carboxylase (PEPC) activity [31], thus lowering the rate of PEP transport into the chloroplast, where PEP, in its dephosphorylated form (pyruvate), feeds into the MEP pathway [81]. Although understanding of the metabolic regulation of the MEP pathway has emerged in the past decade, knowledge about the dynamic change of the regulation steps of the MEP pathway’s response to environmental stimuli, especially nutrient disorders, is limited. Future study should be aimed at a complete understanding of the molecular regulation of the MEP pathway under Mg deficiency as well as other nutrient disorder.

### 3.5. Mg Deficiency Affected the Genes Involved in Lipid Metabolism (GO:0006629)

Lipids are one of the major components of the plasma membrane, which is the interface between the cell and the environment [82]. Available literature showed that environmental stimuli could alter lipid content and lipid metabolism in higher plants. For instance, the content of polar lipid compounds in *Arabidopsis* and *Virgilia divaricata* was decreased by UV-B radiation [80], heat stress [83], P deficiency [84] and high light [14]. Moreover, RNA-Seq and proteomic studies showed that the genes or functional proteins involved in lipid metabolism could be altered by salinity stress in barley [85], low temperatures in cassava [86], P deficiency in *Arabidopsis* [87], boron deficiency in *C. sinensis* [63], and Mg deficiency in *C. reticulata* [19] and *C. sinensis* leaves [88]. Our previous studies showed that the expression levels of gene and protein abundance of phosphatidylinositol 3-kinase and 4-kinase were simultaneously upregulated by Mg deficiency in *C. sinensis* leaves, thus contributing to the Mg-deficiency-tolerance of plants [1,88]. Here, we successfully identified and quantified 194 DEGs related to lipid metabolism in *C. sinensis* leaves, of which, 86 DEGs were upregulated and 108 DEGs were downregulated by Mg deficiency (Table S8).

Phosphoinositide phospholipases C (PI-PLC) are the principle enzymes, which catalyze the initial step of phospholipid breakdown in cell membrane and generate multiple lipid derived second messengers such as diacylglycerol (DAG) and inositol 1,4,5-trisphosphate (IP3), which are involved in a wide range of cellular processes including plant development and stress response [82]. The alternation of PLC gene expression levels by environmental stimuli have been reported in *Arabidopsis* under heat [83], cold [89], dehydration and salt stress [90]. In the current study, Mg deficiency upregulated the expression levels of two *PLC2* genes and downregulated two PLC isoform (*PLC4* and *PLC6*) genes in *C. sinensis* leaves. Vossen et al. [91] also found that the different PLS genes in tomato leaves showed distinct expression patterns in response to pathogen infection. Previous research indicated that under phosphorus-limiting condition, membrane phospholipids provided a pool for inorganic phosphate by the activity of intracellular phospholipase C, which can be used for the synthesis of other essential phosphorus-containing biomolecule in *Sinorhizobium meliloti* [92]. The up-regulation of *PLC2* genes may also be explained partially by this way, as Mg deficiency significantly decreased the P content in *C. sinensis* leaves [93]. Besides PLCs, other form of phospholipase such as patatin-like phospholipase family protein, phospholipase A1-I gamma and phospholipase A1-II gamma, were also differentially regulated by Mg deficiency (Table S8).

Fatty acids (FA), a major source of energy in plants, are the main components of plant membrane lipids. The increasing of membrane unsaturated FA catalyzed by fatty acid desaturase, plays a critical role in ion permeability, appropriate fluidity and stability of membrane [94]. The expression of a fatty acid desaturase gene (stearoyl-ACP desaturase SSI2) was upregulated almost 10 fold under cold stress in cassava apical shoots, implying that this gene may play a role in enhancing membrane fluidity [86]. Here, we report that genes encoding Omega-3 fatty acid desaturase (endoplasmic reticulum) and acyl-acyl-carrier-protein desaturase (chloroplast) and genes encoding the sphingolipid long chain base delta 8 desaturase, temperature-sensitive omega-3 fatty acid desaturase (chloroplast), omega-6 fatty acid desaturase (chloroplast) and omega-6 fatty acid desaturase isozyme 2 (endoplasmic reticulum) were upregulated and downregulated in *C. sinensis* leaves by Mg deficiency, respectively (Table S8). The differentially regulated FA desaturase genes means that fine-tuning of the saturation of fatty acids in different subcellular organelles may confer resistance to Mg deficiency and subsequent side effects downstream in *C. sinensis*, although such resistance could not compensate for the adverse injury as the TBARS content was increased in *C. sinensis* leaves by Mg deficiency (Figure 3I).

Very long chain fatty acids (VLCFAs) are involved in diverse biological functions such as membrane constituent, surface barrier and seed storage compound. The first step of VLCFAs biosynthesis is the condensation of two carbons to an acyl-CoA, which is catalyzed by 3-keto acyl-CoA synthase (KCS) [95]. Here, we found three KCS genes (3-ketoacyl-CoA synthase 11, 17 and 21) were downregulated by Mg deficiency (Table S8). This is consistent with the results obtained from *C. sinensis* under B deficiency [63], rice under Mg deficiency [96], *Arabidopsis* under drought stress [97] and flax (*Linum usitatissimum* L.) under saline–alkaline stress [98]. The down-regulation of KCS genes may indicate that the biosynthesis of VLCFAs was slowed down by adverse conditions including Mg deficiency. Besides KCS genes, the differential regulation of genes encoding acyl-acyl-carrier-protein thioesterase type B, acyl-coenzyme A oxidase, acyl-protein thioesterase 2, lecithin-cholesterol acyltransferase and protein WAX2 (fatty acid hydroxylase domain containing CER1-like protein) by Mg deficiency implied that the balance of fatty acid metabolism might be impaired in *C. sinensis* leaves (Table S8). Interestingly, a gene encoding the probable mannitol dehydrogenase, which catalyzes the conversion of mannitol to mannose, was upregulated by Mg deficiency in *C. sinensis* leaves. Previous research indicated that mannose could induce stomatal closure through methods mediated by ROS production [99]. This is accordant with the higher level of ROS production and stomatal closure represented as the H_2_O_2_ production and stomatal conductance in *C. sinensis* leaves (Figure 3H) [18].

### 3.6. Mg Deficiency Affected the Genes Involved in Metabolic Process (GO: 0008152) and Biological Process (GO: 0008150)

A total of 2113 DEGs were clustered into metabolic process with 1142 DEGs and 971 DEGs upregulated and downregulated by Mg deficiency, respectively, whereas a total of 609 more DEGs were clustered into biological process than into metabolic process. Because some of these DEGs overlapped with those clustered into the GO terms discussed above, hereafter we mainly focus on the DEGs involved in the conspicuously altered pathway that differed with those discussed above.

Plant hormones simultaneously coordinate physiological responses to biotic and abiotic stresses [100]. Here, we found that the expression levels of key genes such as S-adenosylmethionine synthase (*SAMS*), 1-aminocyclopropane 1-carboxylate synthase (*ACCS*) and 1-aminocyclopropane-1-carboxylate oxidase (*ACCO*), which are involved in ethylene biosynthesis pathway, and several ethylene-responsive transcription factors (*ERFs*) were all significantly upregulated by Mg deficiency (Table S9). Available studies show that ethylene interacts with nutrient uptake (e.g., P and N) and controls plant response under growth-limiting condition or stress. Environmental stress could speed up the ethylene production rate in plants [101]. The up-regulation of genes involved in ethylene biosynthesis might imply that Mg deficiency enhances ethylene production of *C. sinensis* leaves, which is consistent with the results obtained in *Arabidopsis* under Mg deficiency [29]. Besides the DEGs related to ethylene, genes encoding indole-3-acetic acid inducible 3, indole-3-acetic acid inducible 9, auxin efflux carrier component, auxin-induced proteins and auxin-responsive proteins, which are involved in auxin response, were also induced by Mg deficiency in *C. sinensis* leaves (Table S9). The up-regulation of transport proteins/genes and adjustment of metabolic processes of auxin may help root hair elongation and enhance nutrient absorption under adverse conditions [66,101]. According to the up-regulation of auxin related genes, we speculate that Mg deficiency might also enhance auxin production in *C. sinensis* leaves.

As we described above, Mg deficiency significantly decreased CO_2_ assimilation and F_v_/F_m_, ET_o_/RC and increased the intercellular CO_2_ concentration and F_o_ in *C. sinensis* leaves (Figure 3A–E). This result is highly concordant with RNA-Seq data, which show that genes encoding chloroplast apparatus such as chloroplast oxygen-evolving complex (OEC)/thylakoid lumenal 25.6 Da protein, chloroplast photosynthetic water oxidation complex 33 kDa subunit, chloroplast ribose-5-phosphate isomerase and photosystem I (PS I) reaction center subunits, thioredoxins (THX), protoporphyrinogen oxidase (PPO) and photosystem II (PS II) core complex proteins were downregulated by Mg deficiency in *C. sinensis* leaves (Table S9). One of the apparent symptoms of Mg deficiency is the thickening, suberification and even splitting of leaf veins due to the accumulation of callose or lignin (Figure 1; Figure 3J). Here, we found that several DEGs encoding cellulose synthase A, callose synthase, laccase and cytochrome P450, which are responsible for the biosynthesis of cellulose, callose or lignin, were upregulated by Mg deficiency in *C. sinensis* leaves (Table S9).

Transcription factors tightly control plant development, nutrient acquisition, biotic and abiotic stresses [102]. Here, we found that the gene expression levels of two AP2, two bHLH and seven MYB containing transcription factors were downregulated, meanwhile, the gene expression levels of five MYB transcription factors, 19 NAC domain-containing proteins and 11 WRKY transcription factors were upregulated in *C. sinensis* leaves (Figure 5; Table S9). This result was in accordance with the research of Hermans et al. [28], who also found that Mg deficiency altered the expression levels of several transcription factors and upregulated the gene expression level of *AP2* in *Arabidopsis*. Recent evidence showed that MYB transcription factor could act as a sensory switch regulating lignin biosynthesis in woody plant cells [103]. The up-regulation of MYB transcription factor may also involve in the accumulation of lignin content in *C. sinensis* leaves under Mg deficiency (Figure 3J). Mg deficiency decreased light energy utilization and phloem loading of photosynthate, thus increased the oxidative stress and the accumulation of photosynthate in source leaf cells [9,22]. As a strategic response, DEGs involved in cell wall remodeling such as xyloglucan endotransglucosylase/hydrolases (*XTH*), and the antioxidant system such as peroxidase (*POD*), *S*-adenosylmethionine-dependent methyltransferase (*SAM-MT*), glycoside hydrolase (*GH*), cytochrome P450 (*CYP450*) were upregulated by Mg deficiency in *C. sinensis* leaves, indicating their probable roles in cell wall loosening and antioxidation under Mg deficiency (Figure 5; Tables S9 and S10) [19]. A previous study showed that Mg deficiency led to leaf cell senescence and necrosis in rice and bean, respectively [104]. Local necrosis may facilitate the infection of pathogen and bacteria, therefore, pathogen and disease resistant related genes such as pathogenesis-related protein 5, pathogenesis-related protein 10, TIR-NBS-LRR-TIR type disease resistance protein, chitinase-like protein 2, late blight resistance protein R1-A and TMV resistance protein N, etc., were upregulated in *C. sinensis* leaves under Mg deficiency (Tables S9 and S10).

Interestingly, Mg deficiency might also affected nutrient absorption and distribution in sub-cellular level by decreasing genes encoding potassium channel AKTs (plasma membrane), K^+^ efflux antiporter 3 (chloroplast), high-affinity potassium transporter protein 1 (plasma membrane) and increasing genes encoding magnesium transporter MRS2-1 (mitochondria), MRS2-3 (mitochondria), magnesium transporter CorA-like family protein (plasma membrane), magnesium and cobalt efflux protein CorC (plasma membrane), ammonium transporter 2 (plasma membrane) [105,106]. Moreover, DEGs encoding Nitrate transporters were differentially affected by Mg deficiency with seven upregulated and nine downregulated in *C. sinensis* leaves (Tables S9 and S10).

## 4. Materials and Methods

### 4.1. Plant Culture and Mg Treatment

Uniform seeds of *C. sinensis* were sown in a plastic tray containing clean river sand and kept moist under natural light and temperature in the greenhouse of Fujian Agriculture and Forestry University. After the seeds germinated, tender seedlings were irrigated with 1/4 strength nutrient solution every other day. Seven weeks later, uniform seedlings with two leaves and one sprout were transplanted to 6 L pottery pots containing clean river sand and irrigated with 500 mL 1/2 strength nutrient solution every two days. There were two seedlings per pot. The full strength nutrient solution contained the following macronutrients (in mM): KNO_3_, 5; Ca(NO_3_)_2_, 5; KH_2_PO_4_, 1; MgSO_4_, 2; micronutrients (in μM): H_3_BO_3_, 10; MnCl_2_, 2; ZnSO_4_, 2; CuSO_4_, 0.5; (NH_4_)_6_Mo_7_O_24_, 0.065; and FeSO_4_-EDTA, 20. Ten weeks after transplanting, each pot was supplied every other day until saturated with Mg-sufficient (control, 1 mM MgSO_4_) or Mg-deficient (-Mg, 0 mM MgSO_4_) nutrient solution for 16 weeks. Sulfur (S) concentration was maintained at a constant level by adding 1 mM Na_2_SO_4_ instead of MgSO_4_. At the end of the experiment, fully-expanded (about 7 weeks old) leaves from different replicates and treatments were collected and used for all the measurements. Leaf discs (0.608 cm^2^) were punched and collected at noon on a sunny day. In the meantime, root apices (about 5 mm) were excised from the same seedlings used for leaf sampling and collected. Leaf samples were wrapped in aluminum foil, immersed in liquid nitrogen and stored at −80 °C until extraction.

### 4.2. Leaf Gas Exchange and Chlorophyll a Fluorescence

Leaf gas exchange was measured by using a CIRAS-2 portable photosynthesis system (PP systems, Amesbury, MA, USA) at an artificial CO_2_ concentration (380 µmol mol^–1^) supplied by a CO_2_ cylinder and a controlled light intensity of 1000 μmol m^−2^ s^−1^ between 10:00 am and 11:30 am on a clear day. During measurements, leaf temperature and vapor pressure deficit were 30.1 ± 0.2 °C and 1.37 ± 0.1 kPa, respectively. Chlorophyll a fluorescence parameters were measured by using Handy PEA parable instrument (Hansatech, Norfolk, UK) on the dark-adapted leaves.

### 4.3. Plant Dry-Weight (DW), Mg and Leaf Soluble Carbohydrates Content

At the end of experiment, ten plants per treatment from different pots were separated into roots, stems and leaves, loaded into kraft bags and oven dried until at a constant weight at 70 °C. After DW was measured by an electronic balance, plant tissues were ground into fine powder. Mg concentration in roots, stems and leaves was measured by atomic absorption spectroscopy after dry ashing and digestion with 1 M HCl [107]. Sucrose, fructose, glucose and starch in leaves and roots were extracted and measured according to the methods described by Yang et al. [9] and Jones et al. [108]. Briefly, about 100 mg samples were extracted three times with 80% (*v*/*v*) ethanol at 80 °C. After the extracts were evaporated, the resulting pellets were thoroughly dissolved with 3 mL ddH_2_O. Glucose was measured in 1 mL reaction mixture of 100 mM inidazole-HCl (pH 7.9), 5 mM MgCl_2_, 0.5 mM NAD, 1 mM ATP, 2 units G6PDH and 2 units hexokinase. Fructose and sucrose was measured at the same reaction solution mentioned above by adding 2 units phosphoglucose isomerase and 20 units invertase, respectively. Starch was determined by enzyme kinetic method as glucose equivalents with G6PDH and hexokinase.

### 4.4. Measurements of H_2_O_2_ Production, TBARS and Lignin Content in C. sinensis leaves

H_2_O_2_ production was measured according to Yang et al. [109]. The content of H_2_O_2_ was calculated with an extinction coefficient of ε = 26.6 cm^−1^ mM^−1^. TBARS was extracted and measured according to the methods described by Hodges et al. [110]. For lignin measurement, leaf samples were homogenized in 95% ethanol. After centrifugation at 1500 *g* for 5 min, the pellet was washed three times with 95% ethanol and twice with ethanol-hexane (1:2, *v*/*v*). The washed pellet was allowed to air-dry. The lignin content was determined according to the method described by Morrison with some modifications [111]. There were four replicates for H_2_O_2_, TBARS and lignin contents.

### 4.5. Total RNA Extraction and RNA-Seq

Samples from five different plants were pooled into one biological replicates that used to RNA extraction and further sequence analysis. There were two biological replicates per treatments. Total RNA were extracted from leaves and roots samples by using TRIzol reagent (Invitrogen, Carlsbad, CA, USA). Degradation and contamination of total RNA were monitored by 1% agarose electrophoresis. The purity and integrity of total RNA were measured by Nanodrop 2000 and Qubit 4 Fluorometer (ThermoFisher, New York, NY, USA). Only the intact RNA samples with the ratio of A260 (absorbance at 260 nm) to A280 between 1.8 and 2.0 were submitted for further analysis. Message RNA (mRNA) was enriched from 3 μg total RNA by using magnetic beads with oligo dT (Qiagen, Valencia, CA, USA). The resulting mRNA was then fragmented into short fragments (200 nt) and used for first-strand cDNA synthesis with random hexamer-primers. Subsequently, second strand cDNA was synthesized by using DNA polymerase I and purified by AMPure XP beads (Beckman Coulter, Beverly, MA, USA). Purified second-strand cDNA were then put through end repair, 3’ end adenosine tailing, sequencing adaptor connection, fragment selection by AMPure XP beads and finally PCR amplification. The library quality was assessed using the Agilent Bioanalyzer 2100 system (Agilent, Santa Clara, CA, USA). After cluster generation, the library amplicons were sequenced on the Illumina HiSeq 4000 platform (Illumina Inc., San Diego, CA, USA) using the paired-end technology in a single run. Illumina GA Pipeline (Version 1.6) was used to perform the original image process to sequences, base calling and quality value calculation, in which 125 bp/150 bp paired-end reads were generated.

### 4.6. RNA Reads Mapping and Analysis of DEGs

Clean reads were obtained by removing adapter reads, reads with more than 10% N (uncertain base) and low quality reads (the proportion of Q20 more than 50%). After getting rid of the reads mapping to ribosomal RNA, the remaining reads were mapped to the *C. sinensis* genome published by Huazhong Agricultural University (http://citrus.hzau.edu.cn/orange/) by TopHat2 software [112]. The gene expression level was calculated by FPKM (Fragments Per Kilobase of transcript per Million mapped reads) method [113]. Genes with an adjusted *p*-value < 0.05 and fold change (based on log_2_) >1 or <−1 identified by DESeq software were considered DEGs. Functional annotation of DEGs was carried out by the method described by Young et al. [33] and Kanehisa et al. [114].

The RNA-seq data was deposited in NCBI database (https://www.ncbi.nlm.nih.gov/sra/) with SRA accession number PRJNA547836.

### 4.7. RT-qPCR Analysis of DEGs

Total RNA extraction and first-strand cDNA synthesis were performed by the methods described above. There were three biological replicates and each replicate contained pooled samples from five different plants (one plant per pot). Gene special primer pairs were designed by Premier Primer 5.0 software (Premier Biosoft International, Palo Alto, CA, USA) according to the sequences published in the citrus genome mentioned above (Table S11). RT-qPCR was carried out in a mixture containing 10 μL Bestar^TM^ qPCR MasterMix SYBR Green (DBI Bioscience, Shanghai, China), 0.4 μL of 10 μM forward primer, 0.4 μL of 10 μM reverse primer, 2 μL cDNA diluted template and 7.2 μL ddH_2_O. RT-qPCR was performed by using a CFX96 Touch^TM^ Deep Well Real-Time PCR Detection System (Bio-Rad, Hercules, CA, USA). The cycling conditions were 30 s at 95 °C, followed by 40 cycles of 95 °C for 10 s, 60 °C for 20 s, 72 °C Cr 20 s. Relative gene expression was calculated by using the ddCt algorithm. For the normalization of different samples and replicates, the polyubiqutin gene (Cs2g20650) was used as an internal standard and the control samples were used as references whose expression levels of each gene were set to 1.

### 4.8. Experimental Design and Statistical Analysis

There were 20 pots for each treatment in a completely randomized design. Experiments were conducted with 3–10 replicates (one plant per replicate). There were ten replicates for plant biomass, five replicates for Mg contents, three replicates for RT-qPCR analysis, four replicates for TBARS and H_2_O_2_ content measurement, respectively. Results were displayed as means ±SD for *n* = 3–10. Means were separated by the student’s *t*-test at *p* < 0.05.

## 5. Conclusions

Mg deficiency decreased CO_2_ assimilation, but increased starch, sucrose, TBARS contents and H_2_O_2_ production in *C. sinensis* leaves. A total of 4864 genes showed differential expression in response to Mg deficiency as revealed by RNA-Seq and the transcriptomic data were further validated by real-time quantitative RT-qPCR. GO enrichment analysis indicated that the mechanisms underlying Mg deficiency tolerance in *C. sinensis* may be attributed to the following aspects: (a) enhancing microtubule-based movement and cell cycle regulation (kinesin, Sugar carrier protein C); (b) elevating signal transduction in response to biotic and abiotic stimuli (*WAK*, *LecLPK*, *SGTA*, etc.); (c) altering biological processes by tightly controlling phosphorylation especially protein phosphorylation (*PK*, *GsSRK*, *LRRPK*, *MAPKKK*, etc.); (d) down-regulating light harvest and photosynthesis process due to accumulation of carbohydrates (*OEC*, *PS II reaction center W*, *PPO*, *THX*, etc.); (e) up-regulating cell wall remodeling and antioxidant system (*XTH*, *POD*, *SAM-MT*, *GH*, *CYP450*, etc.) (The full name of acronyms could be found in Table S12). Our results provide a comprehensive insight into the transcriptomic profile of key components involved in the Mg deficiency tolerance in *C. sinensis* and enrich our understanding of the molecular mechanisms by which plants adapted to Mg deficient condition.

## Figures and Tables

**Table 1:** Basic information of transcriptomic analysis of *C. sinensis* leaves under different Mg treatments.

Libraries	Raw Reads	Clean Reads	Clean Bases	Error Rate (%)	Q20 (%) ^a^	GC Content (%)	Total Mapped (%)	Multiple Mapped (%)	Unique Mapped (%)
Control 1	60,621,646	55,148,298	8.27 G	0.01	97.23	46.58	40,622,047 (73.66%)	1,447,601 (2.62%)	3,9174,446 (71.03%)
Control 2	63,560,528	60,296,780	9.04 G	0.02	96.46	46.48	44,649,336 (74.05%)	1,515,278 (2.51%)	43,134,058 (71.54%)
Mg deficiency 1	46,690,360	44,741,382	6.71 G	0.02	96.48	45.52	34,110,961 (76.24%)	1,256,345 (2.81%)	32,854,616 (73.43%)
Mg deficiency 2	48,980,872	46,926,606	7.04 G	0.02	96.27	45.36	35,701,067 (76.08%)	1,309,337 (2.79%)	34,391,730 (73.29%)

^a^ Q20 means the percentage of bases with a Phred value of more than 20, which was calculated by the formula Phred = −10log_10_(e).

**Table 2:** FPKM distribution of *C. sinensis* transcriptome libraries.

FPKM Interval	Control 1	Control 2	Mg Deficiency 1	Mg Deficiency 2
0~1	15,556 (51.47%)	15,509 (51.31%)	14,070 (46.55%)	14,025 (46.40%)
1~3	3142 (10.40%)	3157 (10.44%)	3038 (10.05%)	3009 (9.96%)
3~15	6197 (20.50%)	6182 (20.45%)	6475 (21.42%)	6545 (21.65%)
15~60	3649 (12.07%)	3694 (12.22%)	4664 (15.43%)	4663 (15.43%)
>60	1682 (5.56%)	1684 (5.57%)	1979 (6.55%)	1984 (6.56%)

FPKM, Fragments Per Kilobase of transcript sequence per Million base pairs sequenced.

## Supplementary Materials

Supplementary materials can be found at https://www.mdpi.com/1422-0067/20/13/3129/s1. Table S1. The downregulated DEGs in *C. sinensis* leaves under Mg deficiency. Table S2. The upregulated DEGs in *C. sinensis* leaves under Mg deficiency. Table S3. Enriched GO terms of DEGs in *C. sinensis* leaves. Table S4. DEGs enriched in microtubule-based movement (GO: 0007018). Table S5. DEGs enriched in carbohydrate binding activities (GO: 0030246). Table S6. DEGs enriched in phosphorus metabolic process (GO:0006793, GO:0016310 and GO:0006468). Table S7. DEGs enriched in isoprenoid, terpenoid and carotenoid metabolic process (GO:0006720, GO:0006721 and GO:0016116). Table S8. DEGs enriched in lipid metabolism (GO:0006629). Table S9. DEGs enriched in metabolic process (GO: 0008152). Table S10. DEGs enriched in biological process (GO: 0008150). Table S11. Primer pairs used for RT-qPCR analysis. Table S12. List of the acronyms of some nouns.
